# A Case of Micro-medullary Thyroid Carcinoma Presenting as Cancer of Unknown Primary

**DOI:** 10.7759/cureus.87085

**Published:** 2025-07-01

**Authors:** Kyosuke Seguchi, Tomohiro Enokida, Toshiki Oba, Akira L Yoshikawa, Nobuhiro Nakatake, Oyama Yu

**Affiliations:** 1 Medical Oncology, Kameda Medical Center, Kamogawa, JPN; 2 Head and Neck Cancer Oncology, National Cancer Center Hospital East, Kashiwa, JPN; 3 Pathology, Nagasaki University Hospital, Nagasaki, JPN; 4 Pathology, Kameda Medical Center, Kamogawa, JPN; 5 Endocrinology, Kameda General Hospital, Kamogawa, JPN

**Keywords:** calcitonin, cancer of unknown primary, cushing’s syndrome, ectopic acth syndrome, medullary thyroid carcinoma, neuroendocrine tumor, ret mutation, selpercatinib, sporadic mtc, targeted therapy

## Abstract

Cancer of unknown primary (CUP) with neuroendocrine features is classified as a favorable subset, and treatment is recommended based on the grade of the neuroendocrine tumor (NET). Although rare, medullary thyroid carcinoma (MTC) can present as CUP with neuroendocrine features and should be carefully considered as a crucial differential diagnosis in such cases. A 65-year-old man with no relevant medical history presented with a gradually enlarging left cervical mass. Laboratory evaluation revealed hypokalemia due to adrenocorticotropic hormone (ACTH)-dependent Cushing’s syndrome. Contrast-enhanced computed tomography (CT) demonstrated multiple lymphadenopathies, hepatic masses, and bilateral pulmonary ground-glass opacities. A biopsy of a cervical lymph node revealed a neuroendocrine tumor grade 2 (NET G2), but the primary tumor was undetectable on imaging. Elevated serum calcitonin and thyroid ultrasound identified a small left thyroid nodule, fine-needle aspiration, and calcitonin immunostaining on the lesion pathologically revealed MTC. Given the rapidly progressive disease, vandetanib was initiated while awaiting molecular testing for rearranged during transfection (RET)-alteration. Following the detection of the RET M918T mutation, treatment was switched to selpercatinib, and rapid tumor response and endocrine symptom resolution were observed. This case highlights the importance of evaluating serum calcitonin, specifically in patients with neuroendocrine carcinoma of unknown primary, where MTC should be considered as a critical differential diagnosis. Thyroid ultrasound should be performed to identify even small lesions of medullary thyroid carcinoma (micro-MTC). Identification of MTC not only leads to targeted therapies that may improve prognosis, but also allows for genetic risk assessment and early intervention in family members when multiple endocrine neoplasia type 2 (MEN2) is suspected.

## Introduction

Cancer of unknown primary (CUP) is defined as a heterogeneous group of metastatic malignancies in which the primary tumor site remains unidentified despite a standardized diagnostic workup. Although comprehensive evaluations -- including imaging and endoscopic procedures -- are routinely performed, they often fail to localize the primary tumor. However, identifying favorable subsets within CUP, such as neuroendocrine tumors (NETs), can guide effective, specific therapies and improve prognosis.

CUP with neuroendocrine features presents a significant diagnostic and therapeutic challenge in clinical oncology. Among these, medullary thyroid carcinoma (MTC) is a rare but clinically significant cancer arising from parafollicular C cells of the thyroid, often secreting calcitonin and occasionally associated with paraneoplastic endocrine syndromes (e.g., via ectopic adrenocorticotropic hormone (ACTH) production), which would be a significant differential diagnosis in some CUP cases. Notably, MTC can present with widespread metastasis despite a small or undetectable primary lesion, referred to as micro-MTC. Conventional imaging, including a computed tomography (CT) scan, may fail to identify the primary thyroid lesion. While rearranged during transfection (RET) gene alterations are found in approximately 90% of MTC cases, they are essential for guiding treatment selection. RET inhibitors, such as selpercatinib, have shown remarkable clinical activity [1]. Furthermore, distinguishing sporadic from hereditary MTC via germline testing is also critical for appropriate familial risk assessment and management.

Here, we present a case of micro-MTC with RET M918T mutation, initially presenting with a CUP with neuroendocrine features and ectopic ACTH production. The diagnosis was made through serum calcitonin testing and thyroid ultrasound, and the disease responded dramatically to selpercatinib following the identification of the RET mutation. This case emphasizes the diagnostic utility of serum calcitonin and thyroid ultrasound and the therapeutic value of molecular testing in CUP with neuroendocrine features.

## Case presentation

**Figure 1:**
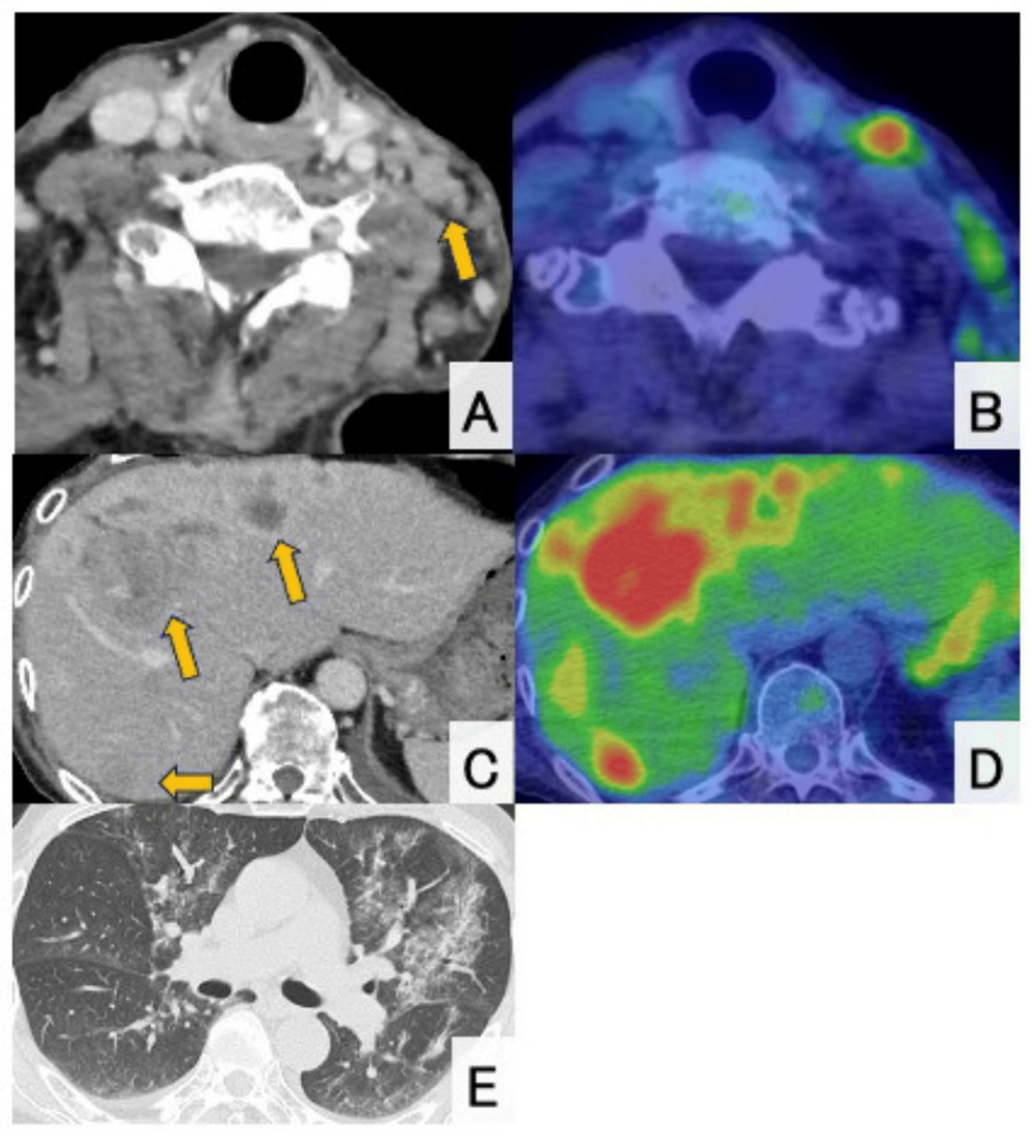
Contrast-enhanced CT and PET findings. (A) and (B) Contrast-enhanced CT and corresponding 18F-FDG-PET/CT images demonstrate multiple enlarged lymph nodes extending from the left cervical region to the axilla, with the largest measuring approximately 32 mm. Notably, no abnormal FDG uptake is observed in the thyroid gland. (C) and (D) Contrast-enhanced CT of the abdomen shows multiple hepatic masses (up to 63 mm) with ring enhancement, which correspond to areas of increased FDG uptake on the PET/CT image. (E) Chest CT shows bilateral ground-glass opacities. PET: positron emission tomography; CT: computed tomography; FDG: fluorodeoxyglucose.

**Figure 2:**
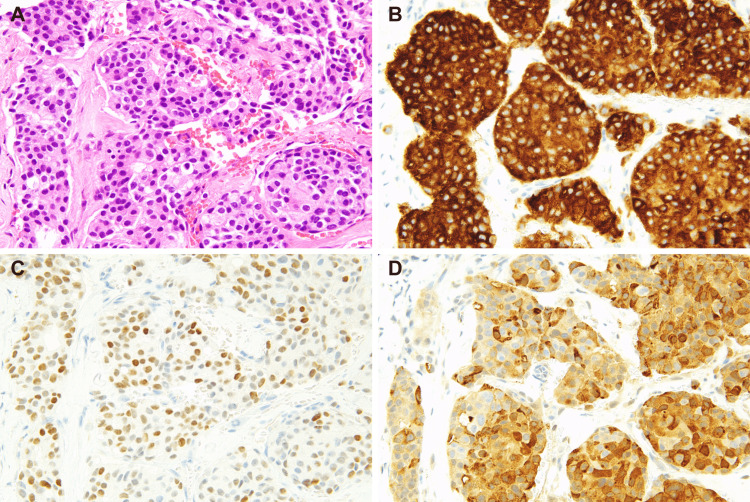
Histological and cytological images of medullary thyroid carcinoma. (A) Hematoxylin and eosin-stained sections of the left cervical tumor reveal a tumor with a cribriform growth pattern. The tumor cells are positive for (B) synaptophysin, (C) thyroid transcription factor-1 (TTF-1), and (D) calcitonin on immunohistochemistry.

**Figure 3:**
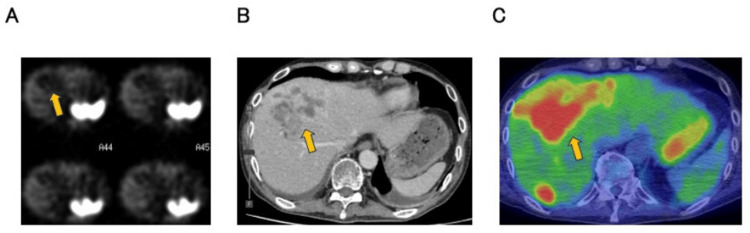
SSTR scintigraphy, contrast-enhanced CT, and FDG-PET. (A) SSTR scintigraphy demonstrated physiological uptake in the spleen and the upper pole of the right kidney, but no abnormal uptake corresponding to the tumor lesions identified on contrast-enhanced CT and FGD-PET.​ (B) Contrast-enhanced CT revealed multiple hepatic masses in S5, characterized by peripheral ring-like enhancement. (C) On PET-CT, intense FDG uptake was observed in the hepatic lesions corresponding to those identified on contrast-enhanced CT. Physiological uptake was also noted in the upper pole of the right kidney. PET: positron emission tomography; CT: computed tomography; FDG: fluorodeoxyglucose; SSTR: somatostatin receptor scintigraphy.

A 65-year-old male with no significant medical history presented with a gradually enlarging left cervical mass over six months and was referred for evaluation on suspicion of a parotid gland malignancy by ultrasound examination at a local clinic. Contrast-enhanced computed tomography (CT) showed multiple lymphadenopathies from the left neck to the axilla (largest measuring 32 mm) and multiple hepatic lesions with ring enhancement (up to 63 mm) (Figures 1A, 1C). Positron emission tomography (PET) and CT scan revealed increased fluorodeoxyglucose (FDG) uptake in the left lateral retropharyngeal space, cervical, supraclavicular, and axillary lymph nodes (SUVmax up to 8.32). Multiple hypodense hepatic masses also demonstrated FDG uptake (SUVmax up to 8.17). However, no abnormal uptake suggestive of a primary tumor elsewhere was observed (Figures 1B, 1D). Upper and lower gastrointestinal endoscopies also could not identify any lesion suggestive of a primary tumor. Histological examination of the biopsied tissue from the cervical tumor revealed carcinoma with solid and cribriform patterns within a dense fibrotic or desmoplastic stroma. The tumor cells exhibited large hyperchromatic nuclei and eosinophilic granular cytoplasm (Figure 2A). The mitotic rate was estimated to be 0.2 mitoses/2 mm². On immunohistochemistry, the tumor cells were positive for CK7, CA19-9, CD56 and synaptophysin (Figure 2B) and negative for p40, p63, CK14, S100, α-SMA, c-kit, androgen receptor, GATA3, HER2 (score 0), HepPar1, maspin, and p53 (wild-type pattern). The Ki-67 index was 7.4% in hot spots. Thus, a neuroendocrine tumor grade 2 was considered one of the most likely diagnoses at this time. However, somatostatin receptor scintigraphy (SSTR) demonstrated no uptake in the hepatic lesions (Figure 3).

Blood examination revealed severe hypokalemia (2.4 mmol/L), attributed to renal potassium loss. Endocrinological assessment confirmed Cushing's syndrome, supported by markedly elevated midnight serum cortisol (65.2 μg/dL), plasma ACTH (862.7 pg/mL), and 24-hour urinary free cortisol (3330 μg/day) (Table 1). Furthermore, cortisol and ACTH levels were not suppressed by the 8 mg dexamethasone suppression test (cortisol 71.2 μg/dL; ACTH 895.4 pg/mL), suggesting ectopic ACTH production. In addition, elevated serum β-D-glucan, in the context of an immunocompromised state due to ectopic ACTH production, and bilateral diffuse ground-glass opacities on chest CT (Figure 1E) were consistent with *Pneumocystis* pneumonia. The diagnosis was confirmed by positive polymerase chain reaction (PCR) testing for *Pneumocystis jirovecii* in bronchoalveolar lavage (BAL) fluid, indicating an immunocompromised state likely secondary to hypercortisolemia associated with Cushing’s syndrome.

**Table 1:** Blood test results were performed on hospital admission. AST: aspartate aminotransferase; ALT: alanine aminotransferase; ACTH: adrenocorticotropic hormone; CEA: carcinoembryonic antigen; NSE: neuron-specific enolase; ProGRP: pro-gastrin-releasing peptide.

Test	Reference range	Results	Unit
Sodium	138-145	138	mEq/L
Potassium	3.6-4.8	2.4	mEq/L
Chloride	101-108	91	mEq/L
Urea	8-20	13	mg/dL
Creatinine	0.65-1.07	0.56	mg/dL
AST	13-30	39	IU/L
ALT	10-42	107	IU/L
Lactate dehydrogenase	124-222	631	IU/L
Total bilirubin	0.4-1.5	0.5	mg/dL
Total protein	6.6-8.1	5.6	g/dL
Albumin	4.1-5.1	2.9	g/dL
C-reactive protein	0.00-0.14	0.71	mg/dL
Glucose	73-109	309	mg/dL
HbA1c	4.9-6.0	8.1	%
White blood cells	3300-8600	9400	/µL
Hemoglobin	13.7-16.8	12.9	g/dL
Platelets	15.8-34.8	9.9	x10^4^/µL
Neutrophils	42.4-75.0	94.3	%
Cortisol	6.24-18.0	65.2	μg/dL
ACTH	8.7-61.5	862.7	pg/mL
CEA	0-5	3816	ng/mL
CA19-9	0-37	159	U/mL
ProGRP	0-81	3611	pg/mL
NSE	0-16.3	73.1	ng/mL
KL-6	105.3-401.2	457.2	U/mL
β-d-Glucan	0-10	366	Pg/ml

**Figure 4:**
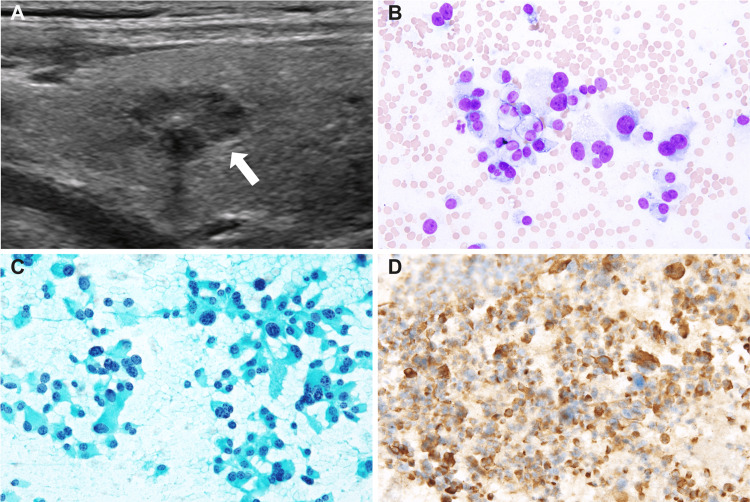
Ultrasound and cytology findings of the thyroid gland. (A) Thyroid US showed a hypoechoic nodule measuring 11 × 6 mm in the left lobe with central calcification. Smears of the thyroid nodule with (B) Giemsa and (C) Papanicolaou staining reveal many large pleomorphic tumor cells with “salt-and-pepper” nuclei. (D) The tumor cells are positive for calcitonin on immunocytochemistry.

Despite a pathological diagnosis of NET G2, the relatively rapid disease progression and markedly elevated serum CEA levels raised clinical suspicion of a mixed neuroendocrine-non-neuroendocrine neoplasm (MiNEN), potentially involving an adenocarcinoma component. However, in light of European Society for Medical Oncology (ESMO) guidelines recommending calcitonin measurement in CUP with neuroendocrine features, serum calcitonin was assessed and revealed a markedly elevated level of 20,200 pg/mL. Furthermore, thyroid ultrasound subsequently identified a hypoechoic nodule measuring 11 × 6 mm in the left lobe with central calcification (Figure 4A), and fine-needle aspiration of this nodule showed remarkably hypercellular smears with loosely cohesive, large pleomorphic tumor cells, the morphology of which appeared identical to those in the left cervical biopsy (Figures 4B, 4C). The cells were positive for calcitonin on immunocytochemistry (Figure 4D). Finally, re-assess of the original cervical lymph node biopsy confirmed a diagnosis of high-grade MTC according to the WHO Classification of Tumors, 5th edition (Figures 2C, 2D).

**Figure 5:**
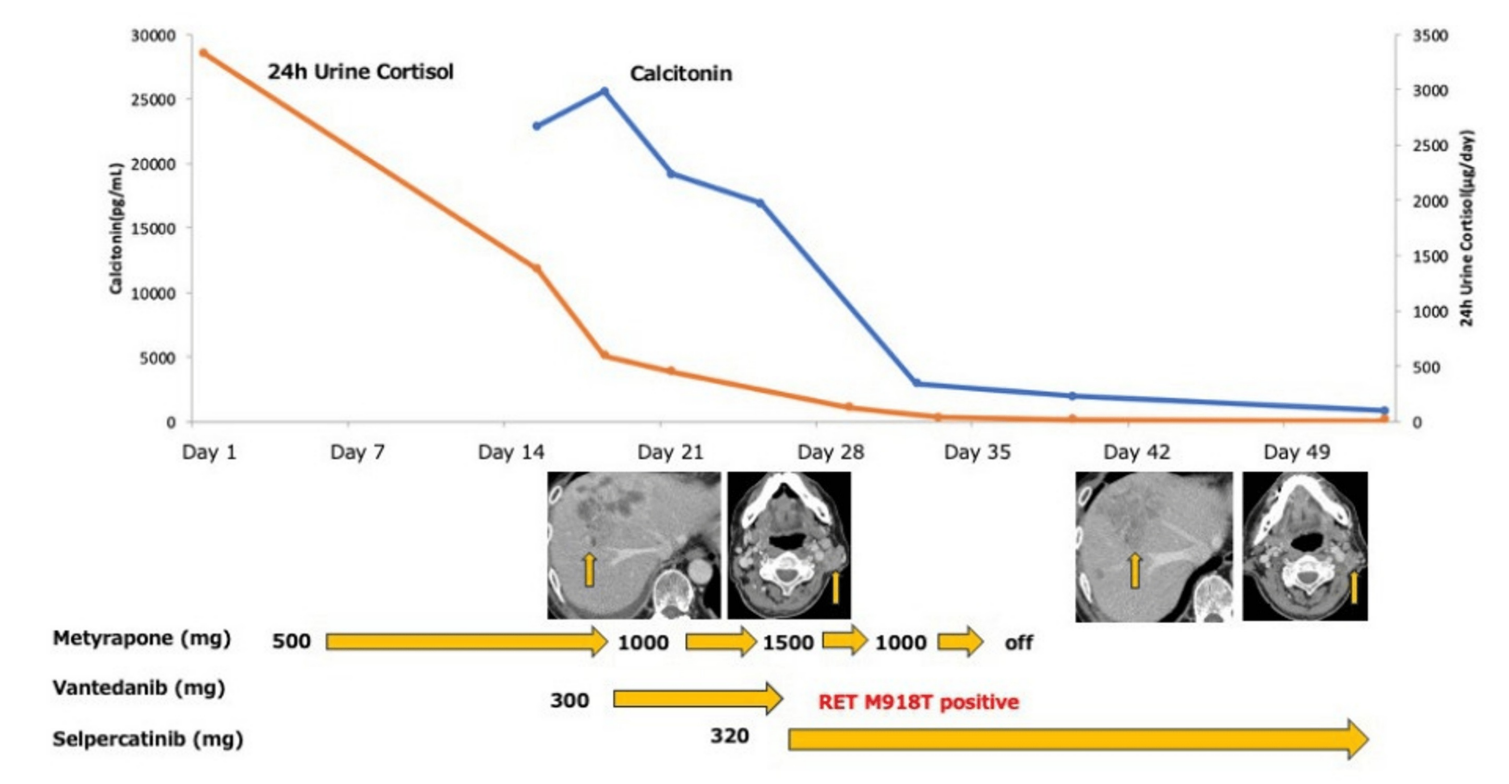
Clinical course. Trends in serum calcitonin (blue line, left axis) and 24-hour urinary cortisol (orange line, right axis) levels are shown over the course of treatment. The patient was initiated on metyrapone (500–1500 mg/day) to control ACTH-dependent hypercortisolemia, followed by vandetanib (300 mg/day) as a bridging therapy while awaiting genetic testing. Upon confirmation of a RET M918T mutation on day six of vandetanib initiation, targeted therapy with selpercatinib (320 mg/day) was commenced, resulting in rapid reductions in both hormonal markers. Metyrapone was terminated by day 35 due to the resolution of cortisol excess. Representative axial CT images obtained before treatment initiation and 28 days after the start of therapy demonstrate a marked reduction in hepatic and cervical tumor size, consistent with treatment response. RET: rearranged during transfection; ACTH: adrenocorticotropic hormone; CT: computed tomography.

Block therapy with metyrapone was initiated to manage ACTH-dependent Cushing’s syndrome. Given the diagnosis of MTC and the patient’s deteriorating general condition along with rapid disease progression, vandetanib, a multikinase inhibitor with non-selective RET activity, was started at a dose of 300 mg daily while awaiting the results of RET genetic testing. Following the initiation of vandetanib, a marked decline in both serum calcitonin and cortisol levels was observed. On day six of treatment, genetic panel testing using the Oncomine Dx Target Test, next-generation sequencing-based companion diagnostic approved by Japan’s Pharmaceuticals and Medical Devices Agency (PMDA) for detecting RET gene alterations in thyroid cancer, identified a RET M918T mutation, prompting a switch to selpercatinib at 320 mg daily, which led to a rapid and marked reduction in cervical lymphadenopathy and serum calcitonin within approximately one week, demonstrating the potent therapeutic efficacy of RET-targeted therapy. Selpercatinib is currently recommended over multikinase inhibitors, such as vandetanib, for RET-altered MTC, based on improved outcomes in both randomized and non-randomized clinical trials, along with a lower incidence of treatment-related adverse events [1]. ACTH production was managed with metyrapone, which was successfully terminated promptly due to the favorable response to selpercatinib. At the first disease assessment, approximately one month after treatment initiation, significant reductions in the size of the left lymphadenopathies and hepatic masses were observed (Figure 5).

In light of the identified RET M918T mutation, clinical and biochemical evaluation for multiple endocrine neoplasia type 2 (MEN2) was conducted. No suspicious mucosal or submucosal nodules suggestive of neuromas were observed in the oral cavity, lips, eyelids, or lower gastrointestinal tract. Plasma metanephrine and normetanephrine levels were within normal limits. Germline RET mutation screening (exons 5, 8, 10, 11, 13-16, including adjacent intronic regions) was negative, suggesting that the case represents a sporadic MTC rather than a familial form associated with MEN2.

## Discussion

This case underscores the importance of considering MTC in the differential diagnosis of CUP with neuroendocrine features. Identifying favorable subsets in CUP is crucial, as specific treatments can significantly improve prognosis [2]. The National Comprehensive Cancer Network (NCCN) guidelines for occult primary cancers recommend that biopsy-confirmed CUP with neuroendocrine features undergo imaging modalities, such as CT, magnetic resonance imaging (MRI), SSTR-PET/CT, and SSTR-PET/MRI, along with endoscopic evaluations, including esophagogastroduodenoscopy (EGD), endoscopic ultrasonography (EUS), and colonoscopy [3].

If these evaluations fail to identify the primary site, initial treatment should be based on the grade of NET. However, some guidelines recommend thorough skin examination and immunohistochemical staining (IHC) for cytokeratin, TTF-1, CDX-2, and calcitonin for differential diagnoses, including paraganglioma, Merkel cell carcinoma, and MTC. Furthermore, in the workup of secretory syndromes associated with neuroendocrine neoplasms, baseline assessment of plasma metanephrines, urinary 5-hydroxyindoleacetic acid (5-HIAA), and serum calcitonin is recommended as a minimum diagnostic evaluation [4]. In the present case, a comprehensive workup including these evaluations was performed, ultimately leading to the diagnosis of MTC. This IHC panel and secretory syndromes workup is particularly crucial when considering the differential diagnosis of CUP with neuroendocrine features. MTC originates from thyroid parafollicular C cells, which exhibit neuroendocrine characteristics. Approximately 0.6% of MTC cases are associated with ectopic ACTH production [5]. Furthermore, MTC can present with lymph nodes and distant metastases, even when the primary tumor is smaller than 1 cm, known as micro-MTC [6]. In cases like ours, conventional imaging methods may fail to detect the primary lesion [7].

MTC is characterized by RET gene abnormalities in 60-90% of cases, making it highly responsive to RET-targeted therapies, which significantly improve prognosis [8]. This is particularly relevant in high-grade MTC, which constitutes approximately 25% of cases and is associated with significantly poorer outcomes compared to low-grade MTC, with 10-year overall survival rates of only 47% versus 91% [9]. The identification of RET mutations in high-grade tumors may, therefore, have critical prognostic and therapeutic implications. Additionally, MTC can be associated with MEN2, underscoring the importance of precise diagnosis not only for the patient but also for family screening and early intervention.

This case highlights the necessity of measuring serum calcitonin in CUP with neuroendocrine features to investigate the possibility of MTC. Furthermore, the rapid implementation of genetic testing is crucial for definitive diagnosis and targeted therapy selection, both of which can dramatically improve patient outcomes. Recent advancements in novel therapies, including peptide receptor radionuclide therapy (PRRT) and Delta-like ligand 3 (DLL3) bispecific T-cell engager (BiTE) therapies, are also promising treatment strategies for neuroendocrine tumors [10,11].

## Conclusions

This case illustrates a diagnostically challenging presentation of micro-MTC manifesting as CUP. Although conventional workups failed to localize the tumor, marked hypercalcitoninemia led to the identification of a subcentimeter thyroid lesion. The rapid tumor response to selpercatinib and normalization of endocrine abnormalities highlight the clinical value of early calcitonin measurement and RET mutation analysis in CUP with neuroendocrine features. Clinicians should consider MTC in the differential diagnosis of CUP with neuroendocrine features, even in the absence of obvious thyroid involvement on imaging, as timely molecular diagnosis and targeted therapy can profoundly impact prognosis. Furthermore, the diagnosis of MTC has broader implications beyond individual treatment, as it can prompt screening for MEN2. This allows for early detection and intervention in at-risk family members, highlighting the significance of integrating genetic testing not only for therapeutic decision-making but also for hereditary cancer risk assessment.
